# Europe's climate leadership in an ‘America first’ era

**DOI:** 10.1016/j.lanepe.2025.101257

**Published:** 2025-03-04

**Authors:** Kim R. van Daalen, Hedi K. Kriit, José Chen-Xu, Jan C. Semenza, Maria Nilsson, Niheer Dasandi, Slava Jankin, Anil Markandya, Josep M. Antó, Joacim Rocklöv, Cathryn Tonne

**Affiliations:** a British Heart Foundation Cardiovascular Epidemiology Unit, Department of Public Health and Primary Care, University of Cambridge, Cambridge, UK; b Heart and Lung Research Institute, University of Cambridge, Cambridge, UK; c Barcelona Supercomputing Center, Barcelona, Spain; d Heidelberg Institute of Global Health, Heidelberg University, Heidelberg, Germany; e Interdisciplinary Center of Scientific Computing, Heidelberg University, Heidelberg, Germany; f Department of Epidemiology and Global Health, Umeå University, Umeå, Sweden; g Barcelona Institute for Global Health (ISGlobal), Barcelona, Spain; h Universitat Pompeu Fabra (UPF), Barcelona, Spain; i School of Government, University of Birmingham, United Kingdom; j BC3 Basque Centre for Climate Change, Bilbao, Spain; k CIBER Epidemiología y Salud Pública (CIBERESP), Madrid, Spain; l The Institute for Data and Artificial Intelligence, University of Birmingham, United Kingdom

Withdrawing from the World Health Organization and the Paris Agreement (again), cutting climate, health and development funding, purging climate mentions from websites, muzzling the Centers for Disease Control and Prevention, placing Environmental Protection Agency staff on administrative leave, declaring a “*national energy emergency*” with the promise to “*drill, baby, drill*,” - since resuming office, Trump has signed over 30 executive orders (and counting) that reverse climate and health progress. These actions unleash U.S. fossil fuel production and deliver a significant blow to global climate and health efforts.^1^

This comes against a backdrop of 2024 being declared the hottest year on record - the first to surpass the 1.5 °C threshold^2^ - and with the U.S. still reeling from the devastating wildfires that engulfed Los Angeles and the trail of destruction left by hurricanes along its coastal areas months earlier. Worldwide, greenhouse gas levels continue to rise, pushing planetary boundaries, and leaving communities to brace themselves for the impacts of ferocious extreme events. Yet, ten years after pledging to limit global temperature rise to below 2 °C, current mitigation policies around the world remain inadequate, as evidenced by a projected 3.2 °C increase by 2100.^3^ Such temperature increases may even be higher if nations weaken climate action. Whilst many of Trump's actions face legal challenges, the resulting geopolitical turmoil fuelled by the U.S.'s climate scepticism underscores the urgent need for coordinated global climate action like never before.

Since climate change knows no borders, U.S.'s climate inaction, like inaction of any major emitter, will have far-reaching and rippling consequences - disproportionately affecting the well-being of the world's most vulnerable populations. Abandoning efforts to reduce emissions will make the air dirtier, increase the incidence and geographic range of countless diseases, and leave millions of people with worsening health globally. Spain's 2024 simultaneous battle with deadly floods and severe droughts is a stark reminder that climate change already endangers lives in Europe. From food and water insecurity to the spread of infectious diseases; negative health trends are accelerating and expected to worsen under all emission scenarios.^4^ These adverse health outcomes are evident across Europe (Fig. 1), where temperatures rise twice the global rate.^5^ For example, record-breaking summer temperatures in Europe resulted in increased heat-related mortality, with 61,000 and 47,000 excess deaths in 2022 and 2023, alone.^6^

**Fig. 1:**
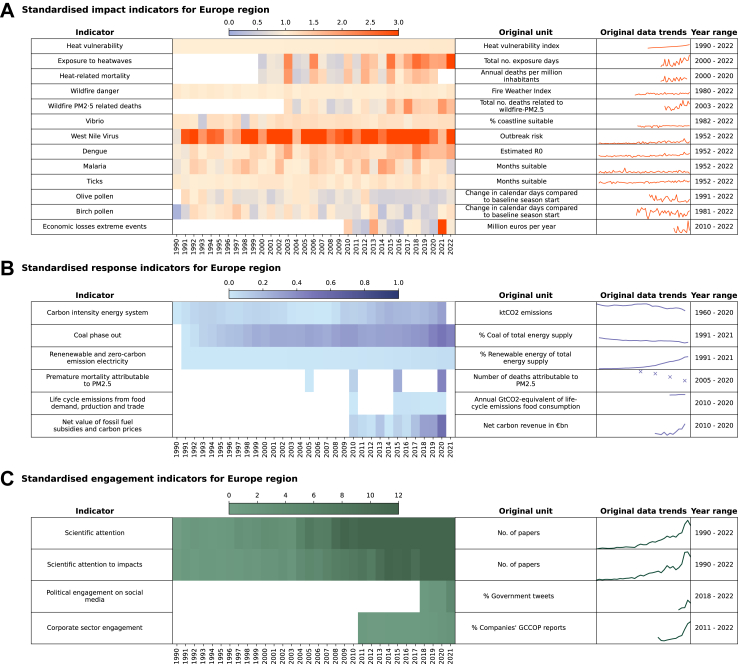
**Overview of standardised impacts, responses and engagement indicators on climate change and health, tracked in the 2024 European report of the *Lancet* Countdown.** (**A**) Climate-related health impact indicators, with stabilisation or worsening of most of most indicators tracked compared to their baseline. (**B**) Climate change response indicators, with some improvement in most indicators compared to their baseline. (**C**) Climate and health engagement indicators, with an increase in engagement across most indicators compared to their baseline. **Notes:** Impact and engagement indicators were standardised by $yearly\;score\;R=\frac{yearly\;value}{base\;value}$. Response (adaptation and mitigation) indicators were standardised by $yearly\;score\;R=\frac{yearly\;value-worse\;case\;value\;}{target\;value-worse\;case\;value}$. The heatmap (1990–2022) represents indicators standardised to a European baseline specified for each indicator. For (**A**), higher standardised values correspond to a worsening of the indicator, and for (**B**) and (**C**) higher standardised values correspond to an improvement of the indicator. The sparkline represents the indicator data trend in its original unit and time coverage (see year range). The y-axes of the sparkline are scaled to the max and min values. Europe is defined following the European Environment Agency (EEA) member and cooperating countries. See the Supplementary Appendix for details on the methodology.

With the U.S. stepping back from emission reductions - and encouraging other nations to follow suit - Europe must stay committed to bold, and decisive climate leadership. Despite facing varied opposition - ranging from economic competitiveness and industrial policy concerns to unknowns about implementation costs and timelines, and resistance from climate-sceptic populists - Europe has expanded renewables use and improved energy efficiency. In 2024, solar power surpassed coal as leading energy source, while natural gas production continues to decline. These decarbonisation efforts enhance energy security, reduce import reliance, and improve public health through decreased air pollution.^5^ As one of the largest economies, the EU can accelerate climate action globally by establishing bilateral agreements and partnerships supporting green transition. Examples include Green Alliances with Canada and Japan, and the Just Energy Transition Partnerships, which highlight the strengths of a unified approach in tackling this global threat to population health.^7^

Importantly, most other industrialised countries - facing the same market forces - have already signalled their continued commitment to the Paris Agreement.^8^ The 2025 Nationally Determined Contributions offer a vital opportunity for the EU and European nations to strengthen their Green Deal ambitions and improve National Adaptation Plans and other key climate policies. This effort should recognise the often-overlooked health co-benefits of climate action and address disparities in climate-related health impacts and adaptive capacity across Europe.^5^

The new 2025 European Commission's work programme appears to threaten ambition towards the green transition in favour of economic competitiveness and “*simplification*” of legislation.^9^ Whilst ambitious, the proposed 90% emissions reduction target for 2040 falls short of slashing Europe's emissions sufficiently in line with fair-share emissions responsibilities (calculated using Europe's population and historical emissions), and meeting targets is critically dependent on policy implementation by countries.^10^ This is not the time for dilution or to be paralysed by the challenges of the emerging geopolitical landscape. Climate action will not only secure energy independence, competitiveness, and help avert some of the most severe impacts on the planet and human health; it will also deliver immediate health and broader societal co-benefits (e.g., reduced air pollution, increased physical activity, and dietary change). Europe must strengthen its long-term commitment to the Paris Agreement and other policies to safeguard the climate and human well-being.

With decisive, equitable action, Europe can lead by example in catalysing health-centred climate policy for the well-being of all its populations.

## Contributors

KRvD, HKK, JCX, and CT conceived the presented commentary, with support from all other authors. KRvD, HKK, and JCX wrote the first draft of the manuscript. KRvD and HKK wrote the Supplementary Appendix and generated the visualisations. Indicators included in the overview figures are generated by the 2024 Lancet Countdown in Europe collaborators, as listed in the Supplementary Appendix. All authors contributed intellectually to the final manuscript.

## Declaration of interests

The authors have no conflicts of interest to declare.
