# Supplementary material for: Europe's climate leadership in an ‘America first’ era
Source: Lancet Reg Health Eur. 2025 Mar 4;51:101257. doi: 10.1016/j.lanepe.2025.101257 (PMC11925515; doi:10.1016/j.lanepe.2025.101257)
Supplement: Supplementary Appendix [file mmc1.docx]

**Supplementary appendix**

This commentary has been written on behalf of the Lancet Countdown in Europe collaboration. The full author list can be found below, ordered following the 2024 Europe Report of the Lancet Countdown.[^1^](https://paperpile.com/c/Y4RsIc/waplv)

Kim R van Daalen, PhDᵃᵇ, Prof Cathryn Tonne, ScDᶜᵈᵉ, Prof Jan C Semenza, PhDᶠ, Prof Joacim Rocklöv, PhDᶠᵍⁱ, Prof Anil Markandya, PhDᵏ, Niheer Dasandi, PhDˡ, Prof Slava Jankin, PhDˡ, Hicham Achebak, PhDᶜᵐ, Joan Ballester, PhDᶜ, Hannah Bechara, PhDⁿ, Thessa M Beck, MScᶜᵈ, Max W Callaghan, PhDᵒ, Bruno M Carvalho, PhDᵃ, Jonathan Chambers, PhDᵖ, Marta Cirah Pradas, MScᶜᵈᵉ, José Chen,ᶜᵈᵉ PhD, Orin Courtenay, PhDᑫ, Shouro Dasgupta, PhDʳˢ, Matthew J Eckelman, PhDᵗ, Zia Farooq, MSci, Peter Fransson, PhDᶠᵍ, Elisa Gallo, PhDᶜ, Olga Gasparyan, PhDᵘ, Nube Gonzalez-Reviriego, PhDᵃᵛ, Prof Ian Hamilton, PhDʷ, Risto Hänninen, DScᵃᵃ, Charles Hatfield, MUPᶠʰ, Kehan He, PhDˣ, Aleksandra Kazmierczak, PhDᵃᵇ, Vladimir Kendrovski, PhDᵃᶜ, Harry Kennard, PhDᵃᵈ, Gregor Kiesewetter, PhDᵃᵉ, Rostislav Kouznetsov, PhDᵃᵃ, Hedi Katre Kriit, PhDᶠᵍ, Alba Llabrés-Brustenga, PhDᵃ, Simon J Lloyd, PhDᶜ, Martín Lotto Batista, MScᵃᶠ, Carla Maia, PhDᵃᵍ, Prof Jaime Martinez-Urtaza, PhDᵃʰ, Zhifu Mi, PhDˣ, Carles Milà, MScᶜᵈ, Jan C Minx, PhDᵒ, Prof Mark Nieuwenhuijsen, PhDᶜᵈᵉ, Julia Palamarchuk, PhDᵃᵃ, Dafni Kalatzi Pantera, PhDᵐ, Marcos Quijal-Zamorano, MScᶜᵈ, Peter Rafaj, PhDᵃᵉ, Prof Elizabeth J Z Robinson, PhDˢ, Nacho Sánchez-Valdivia, MScᶜ, Daniel Scamman, EngDʸ, Oliver Schmoll, Dipl.-Ingᵃᶜ, Maquins Odhiambo Sewe, PhDⁱ, Jodi D Sherman, MDᵃⁱ, Pratik Singh, MScᶠ, Elena Sirotkina, MAᵃʲ, Henrik Sjödin, PhDᶠⁱ, Prof Mikhail Sofiev, PhDᵃᵃ, Balakrishnan Solaraju-Murali, PhDᵃ, Prof Marco Springmann, PhDᵃᵏᵃˡ, Marina Treskova, PhDᶠᵍⁱ, Joaquin Triñanes, PhDᵃᵐ, Eline Vanuytrecht, PhDᵃᵇ, Fabian Wagner, PhDˣ, Maria Walawender, PhDᶻ, Laura Warnecke, PhDᵃᶠ, Ran Zhang, MScᵃⁿ, Marina Romanello, PhDᶻ, Prof Josep M Antó, PhDᶜᵈᵉ, Prof Maria Nilsson, PhDʲ, Prof Rachel Lowe, PhDᵃᵏᵃᵒ

**a** Barcelona Supercomputing Center (BSC), Barcelona, Spain

**b** British Heart Foundation Cardiovascular Epidemiology Unit, Department of Public Health and Primary Care, University of Cambridge, Cambridge, UK

**c** Barcelona Institute for Global Health (ISGlobal), Barcelona, Spain

**d** Universitat Pompeu Fabra (UPF), Barcelona, Spain

**e** CIBER Epidemiología y Salud Pública (CIBERESP), Barcelona, Spain

**f** Heidelberg Institute of Global Health, Heidelberg University, Heidelberg, Germany

**g** Interdisciplinary Center of Scientific Computing, Heidelberg University, Heidelberg, Germany

**h** Heidelberg Institute for Geoinformation Technology (HeiGIT), Heidelberg University, Heidelberg, Germany

**i** Department of Public Health and Clinical Medicine, Umeå University, Umeå, Sweden

**j** Department of Epidemiology and Global Health, Umeå University, Umeå, Sweden

**k** BC3 Basque Centre for Climate Change, Bilbao, Spain

**l** School of Government, University of Birmingham, Birmingham, UK

**m** Institut National de la Santé et de la Recherche Médicale (Inserm), Paris, France

**n** Data Science Lab, Hertie School, Berlin, Germany

**o** Mercator Research Institute on Global Commons and Climate Change (MCC), Berlin, Germany

**p** Energy Efficiency Group, Institute for Environmental Sciences (ISE), University of Geneva, Geneva, Switzerland

**q** The Zeeman Institute and School of Life Sciences, University of Warwick, Coventry, UK

**r** Centro Euro-Mediterraneo sui Cambiamenti Climatici (CMCC), Venice, Italy

**s** Grantham Research Institute on Climate Change and the Environment, London School of Economics and Political Sciences, London, UK

**t** Department of Civil and Environmental Engineering, Northeastern University, Boston, MA, USA

**u** Department of Political Science, Florida State University, Tallahassee, FL, USA

**v** European Centre for Medium-Range Weather Forecast (ECMWF), Bonn, Germany

**w** Energy Institute, University College London, London, UK

**x** The Bartlett School of Sustainable Construction, University College London, London, UK

**y** Institute for Sustainable Resources, University College London, London, UK

**z** Institute for Global Health, University College London, London, UK

**aa** Finnish Meteorological Institute (FMI), Helsinki, Finland

**ab** European Environment Agency (EEA), Copenhagen, Denmark

**ac** European Centre for Environment and Health, WHO Regional Office for Europe, Bonn, Germany

**ad** Center on Global Energy Policy, Columbia University, New York, NY, USA

**ae** Pollution Management Research Group, Energy, Climate, and Environment Program, International Institute for Applied Systems Analysis, Laxenburg, Austria

**af** Medical School of Hannover, Hannover, Germany

**ag** Global Health and Tropical Medicine (GHTM), Associate Laboratory in Translation and Innovation Towards Global Health (LA-REAL), Instituto de Higiene e Medicina Tropical (IHMT), Universidade Nova de Lisboa, UNL, Lisboa, Portugal

**ah** Department of Genetics and Microbiology, Universitat Autònoma de Barcelona, Barcelona, Spain

**ai** Yale University School of Medicine, Yale University, New Haven, CT, USA

**aj** Department of Political Science, The University of North Carolina, Chapel Hill, NC, USA

**ak** Centre for Climate Change and Planetary Health, London School of Hygiene and Tropical Medicine (LSHTM), London, UK

**al** Environmental Change Institute, University of Oxford, Oxford, UK

**am** Department of Electronics and Computer Science, Universidade de Santiago de Compostela, Santiago, Spain

**an** University of Mannheim, Mannheim, Germany

**ao** Catalan Institution for Research and Advanced Studies (ICREA), Barcelona, Spain

**Methodology overview figures**

The overview figures present a selected set of standardised indicators from the 2024 Lancet Countdown in Europe report[^1^](https://paperpile.com/c/Y4RsIc/waplv), which have annual time-series data available. Indicators are divided into climate-related health impacts (**Table 2**), climate change responses (adaptation, mitigation and economics indicators; **Table 3**), and climate-health engagement (**Table 4**). Standardisation has been applied to a European level. Time-series with negative values were transformed by adding a constant so all values were positive before standardisation.

Impact and engagement indicators are standardised by:

$$yearly score =\frac{yearly value}{base value}$$

Response indicators are standardised by:

$$yearly score = \frac{yearly value - worse case value}{target value - worse case value}$$

The following definitions are applied:

**Yearly value** = the annual original value (in the original unit) reported for each indicator.

**Yearly score** = the standardised score for each indicator.

*Impact and engagement indicators*

**Unit** = the unit utilised in the original indicator (as reported within the 2024 Lancet Countdown in Europe report).

**Base period** = the time frame used as a reference for the standardisation.

**Base value** = the value of the original indicator values over the base period.

**Time coverage** = the total time frame the original indicator has data available for.

*Response indicators*

**Worst case value** = the worst value reported over the time period measured of the original indicator.

**Worse case year** = the year the worse case value of the original indicator occurred.

**Target value** = the desired value for each original indicator.

Europe is defined following the definition of the European Environment Agency (EEA), with sub-regions following the UN-Geoscheme (**Table 1**).

##### **Table 1 European Environment Agency (EEA) member and cooperating countries by sub-region.**

| **Central-eastern Europe** | | | |
| --- | --- | --- | --- |
| Bulgaria | Czechia | Hungary | Poland |
| Ukraine | Romania | Slovakia |  |
| **Northern Europe** | | | |
| Denmark | Estonia | Finland | Iceland |
| Ireland | Latvia | Lithuania | Norway |
| Sweden |  |  |  |
| **Southern Europe** | | | |
| Albania (Co) | Bosnia and Herzegovina (Co) | Croatia | Cyprus |
| Greece | Italy | Kosovo* | Montenegro (Co) |
| Serbia (Co) | North Macedonia (Co) | Portugal | Slovenia |
| Spain | Türkiye |  |  |
| **Western Europe** | | | |
| Austria | Belgium | France | Germany |
| Liechtenstein | Luxembourg | Monaco | Netherlands (Kingdom of the)± |
| Switzerland |  |  |  |

##### ** Designation is without prejudice to position on status and is in line with United Nations Security Council resolution (UNSCR) 1244 and the ICJ Opinion on the Kosovo Declaration of independence.*

*± Unless stated otherwise, this report only includes the country of the Netherlands and excludes the Caribbean part of the Netherlands (Kingdom of the). Therefore, the main report refers to “the Netherlands” instead of “the Netherlands (Kingdom of the)”.*

*Co refers to cooperating countries of the EEA.*

Data visualisation was conducted in Python version 3.10.9 using matplotlib, numpy, pandas, and seaborn.

#####

##### **Table 2 Standardisation impact indicators.**

| **Indicator** | **Contributor(s)** | **Coverage** | **Unit** | **Base per** | **Eur base value** |
| --- | --- | --- | --- | --- | --- |
| 1.1.1 Vulnerability to heat exposure | Maquins Odhiambo Sewe,  Joacim Rocklöv | 1990–2022 | Heat vulnerability index | 1990-94 | 37.97 |
| 1.1.2 Exposure of at-risk populations to heatwaves | Jonathan Chambers | 2000-2021 | Total no. exposure days | 2000-04 | 653,393,800 |
| 1.1.4 Heat-related mortality[^2^](https://paperpile.com/c/Y4RsIc/GotdN) | Elisa Gallo  Hicham Achebak,  Joan Ballester. | 2003-2020 | Annual deaths per million inhabitants | 2000-04 | 43.92 |
| 1.2.1 Wildfire danger[^3^](https://paperpile.com/c/Y4RsIc/CLfaf) | Rostislav Kouznetsov,  Risto Hänninen,  Mikhail Sofiev | 1980-2022 | Fire Weather Index | 1980-89 | 17.05 |
| 1.2.1 Wildfire-PM_2.5_-related deaths[^4^](https://paperpile.com/c/Y4RsIc/AQyAC) | Carles Milà,  Cathryn Tonne | 2003-2021 | Total no. deaths related to wildfire-PM_2·5_ | 2003-07 | 466.1 |
| 1.3.1 Climatic suitability for Vibrio | Jaime Martinez-Urtaza, Joaquin Triñanes,  Jan C. Semenza | 1982-2022 | % Coastline suitable | 1982-91 | 19.81 |
| 1.3.2 Climatic suitability of West Nile virus[^5^](https://paperpile.com/c/Y4RsIc/dBxI6) | Zia Farooq,  Jan C. Semenza,  Joacim Rocklöv | 1951-2022 | Outbreak risk | 1951–60 | 0.017 |
| 1.3.3 Climatic suitability for dengue, chikungunya, and Zika[^6^](https://paperpile.com/c/Y4RsIc/d9May) | Pratik Singh, Henrik Sjödin,  Joacim Rocklöv,  Peter Fransson | 1951-2022 | Estimated R0 | 1951–60 | 0.093 |
| 1.3.4 Climatic suitability for malaria[^7^](https://paperpile.com/c/Y4RsIc/uY1c3) | Martín Lotto-Batista,  Rachel Lowe | 1951-2022 | Months climatically suitable | 1951–60 | 0.405 |
| 1.3.6 Climatic suitability for ticks[^8^](https://paperpile.com/c/Y4RsIc/atgsE) | Martín Lotto-Batista,  Rachel Lowe | 1951-2022 | Months climatically suitable | 1951–60 | 4.288 |
| 1.4.1 Allergenic trees (Pollen)[^9^](https://paperpile.com/c/Y4RsIc/jAp15) | Julia Palamarchuk,  Mikhail Sofiev | 1990-2022 | Calender days season start | 1990-94 | 24.60 |
| 1.4.1 Allergenic trees (Birch)[^9^](https://paperpile.com/c/Y4RsIc/jAp15) | Julia Palamarchuk,  Mikhail Sofiev | 1980-2022 | Calender days season start | 1980-84 | 22.40 |
| 4.1.1 Economic losses due to climate-related extreme events | Daniel Scamman | 2010-2022 | Million euros per year | 2010-14 | 18,992 |

#####

##### **Table 3 Standardisation response indicators.**

| **Indicator** | **Coverage** | **Contributor(s)** | **Unit** | **Target value** | **Europe worst value** | **Worst year** |
| --- | --- | --- | --- | --- | --- | --- |
| 3.1.1 Carbon intensity energy system | 1990-2021 | Harry Kennard,  Ian Hamilton | ktCO_2_ emissions | 0 | 1991 | 2016.37 |
| 3.1.2 Coal phase-out | 1991-2021 | Harry Kennard,  Ian Hamilton | % Coal of total energy supply | 0 | 1991 | 25.18 |
| 3.1.3 Renenewable and zero-carbon emission electricity | 1991-2021 | Harry Kennard,  Ian Hamilton | % Renewable energy of total energy supply | 100 | 1991 | 0.32 |
| 3.2.1 Premature mortality attributable to PM_2.5_ | 2005-2020 | Gregor Kiesewetter,  Cathryn Tonne | Number of deaths attributable to PM_2.5_ | 0 | 2005 | 2295 |
| 3.4.1 Life cycle emissions from food demand, production and trade | 2010-2020 | Marco Springmann | GtCO2-equivalent of life-cycle emissions food consumption | 0 | 2020 | 1870.57 |
| 4.2.1 Net value of fossil fuel subsidies and carbon prices | 2010-2022 | Daniel Scamman | Net carbon revenue in €bn | 0 | 2014 | 2.80 |

##### **Table 4 Standardisation engagement indicators**

| **Indicator** | **Contributor(s)** | **Coverage** | **Unit** | **Base period** | **Europe base value** |
| --- | --- | --- | --- | --- | --- |
| 5.1.1 Coverage of health and climate change in scientific articles[^10^](https://paperpile.com/c/Y4RsIc/nbXlN) | Max Callaghan,  Jan Minx | 1990-2022 | No. articles | 1990-1994 | 6.8 |
| 5.1.2 Coverage of the health impacts of anthropogenic climate change[^10^](https://paperpile.com/c/Y4RsIc/nbXlN) | Max Callaghan,  Jan Minx | 1990-2022 | No. articles | 1990-1994 | 12.6 |
| 5.3.2 Political engagement with health and climate change on social media | Hannah Bechara,  Dafni Kalatzipantera | 2018-2022 | % Government tweets | 2018 | 0.026% |
| 5.4 Corporate sector engagement with health and climate change | Ran Zhang,  Dafni Kalatzipantera | 2011-2022 | % GCCOP reports | 2011 | 1.52% |

**References**

1 [van Daalen KR, Tonne C, Semenza JC, *et al.* The 2024 Europe report of the Lancet Countdown on health and climate change: unprecedented warming demands unprecedented action. *Lancet Public Health* 2024; **9**: e495–522.](http://paperpile.com/b/Y4RsIc/waplv)

2 [Ballester J. europe_summer_2022_heat: Heat-related mortality in Europe during the summer of 2022. Github](http://paperpile.com/b/Y4RsIc/GotdN) <https://github.com/BallesterJoan/europe_summer_2022_heat> [(accessed Oct 26, 2024).](http://paperpile.com/b/Y4RsIc/GotdN)

3 [Hänninen R, Sofiev M, Uppstu A, Kouznetsov R. Daily surface concentration of fire related PM2.5 for 2003-2022, modelled by SILAM CTM when using the MODIS satellite data for the fire radiative power. Finnish Meteorological Institute.](http://paperpile.com/b/Y4RsIc/CLfaf) <https://fmi.b2share.csc.fi/records/722bb9d1937548908d2b6c1cfd9f8e5d> [(accessed Oct 26, 2024).](http://paperpile.com/b/Y4RsIc/CLfaf)

4 [Milà C. LCDE2024_wildfires: Wildfire smoke indicators for the Lancet Countdown Europe 2024 edition. Github](http://paperpile.com/b/Y4RsIc/AQyAC) <https://github.com/carlesmila/LCDE2024_wildfires> [(accessed Oct 26, 2024).](http://paperpile.com/b/Y4RsIc/AQyAC)

5 [Farooq Z. LancetEurope_WNV: This repository contains the scripts to reproduce the results for Lancet Europe -West Nile virus indicator. Github](http://paperpile.com/b/Y4RsIc/dBxI6) <https://github.com/Ziaf021/LancetEurope_WNV> [(accessed Oct 26, 2024).](http://paperpile.com/b/Y4RsIc/dBxI6)

6 [Singh P. Dengue-transmission-suitability-LCDE2024. Github](http://paperpile.com/b/Y4RsIc/d9May) <https://github.com/Pratik697/Dengue-transmission-suitability-LCDE2024> [(accessed Oct 26, 2024).](http://paperpile.com/b/Y4RsIc/d9May)

7 [Batista ML. Global Health Resilience / lcde-malaria. GitLab.](http://paperpile.com/b/Y4RsIc/uY1c3) <https://earth.bsc.es/gitlab/ghr/lcde-malaria> [(accessed Oct 26, 2024).](http://paperpile.com/b/Y4RsIc/uY1c3)

8 [Batista ML. Global Health Resilience / lcde-ticks. GitLab.](http://paperpile.com/b/Y4RsIc/atgsE) <https://earth.bsc.es/gitlab/ghr/lcde-ticks> [(accessed Oct 26, 2024).](http://paperpile.com/b/Y4RsIc/atgsE)

9 [Silam-model: Silam model code (mirror of internal FMI svn repo). Github](http://paperpile.com/b/Y4RsIc/jAp15) <https://github.com/fmidev/silam-model> [(accessed Oct 26, 2024).](http://paperpile.com/b/Y4RsIc/jAp15)

10 [Callaghan M. MCC APSIS / lancet-countdown. GitLab.](http://paperpile.com/b/Y4RsIc/nbXlN) <https://gitlab.pik-potsdam.de/mcc-apsis/lancet-countdown/-/tree/main?ref_type=heads> [(accessed Oct 26, 2024).](http://paperpile.com/b/Y4RsIc/nbXlN)
